# Hyaluronan-Based Grafting Strategies for Liver Stem Cell Therapy and Tracking Methods

**DOI:** 10.1155/2019/3620546

**Published:** 2019-07-01

**Authors:** Lorenzo Nevi, Samira Safarikia, Sabina Di Matteo, Francesca Biancaniello, Michele Francesco Chiappetta, Vincenzo Cardinale

**Affiliations:** ^1^Department of Translational and Precision Medicine, Sapienza University of Rome, Viale dell'Università 37, 00185 Rome, Italy; ^2^Medical-Surgical and Biotechnologies Sciences, Polo Pontino, Sapienza University of Rome, Corso della Repubblica 79, 04100 Latina, Italy

## Abstract

Cell adhesion is essential for survival, it plays important roles in physiological cell functions, and it is an innovative target in regenerative medicine. Among the molecular interactions and the pathways triggered during cell adhesion, the binding of cluster of differentiation 44 (CD44), a cell-surface glycoprotein involved in cell-cell interactions, to hyaluronic acid (HA), a major component of the extracellular matrix, is a crucial step. Cell therapy has emerged as a promising treatment for advanced liver diseases; however, so far, it has led to low cell engraftment and limited cell repopulation of the target tissue. Currently, different strategies are under investigation to improve cell grafting in the liver, including the use of organic and inorganic biomatrices that mimic the microenvironment of the extracellular matrix. Hyaluronans, major components of stem cell niches, are attractive candidates for coating stem cells since they improve viability, proliferation, and engraftment in damaged livers. In this review, we will discuss the new strategies that have been adopted to improve cell grafting and track cells after transplantation.

## 1. Introduction

Cell adhesion plays a pivotal role in maintaining the physiologic functions of cells in solid organs, contributing to cellular organization and structure, proliferation, survival, and differentiation. Cell adhesion molecules (CAMs), a family of transmembrane proteins, are involved in cell-to-cell adhesion and in the interaction between cells and the extracellular matrix (ECM) [1, 2]. CAMs are generally characterized by three conserved domains: an intracellular domain that interacts with the cytoskeleton, a transmembrane domain that crosses the lipid bilayers of the cell membrane, and an extracellular domain that interacts either with the same CAMs by homophilic binding or with the ECM by heterophilic binding [3, 4]. The modulation of cell adhesion is a key issue in regenerative medicine [5].

Although tissue engineering has so far aimed at reconstructing organs and tissues or recellularizing natural biomatrices, recently, cell therapy of solid organs has attracted the interest of many scientists and led to promising results in several clinical trials [6–22]. However, the uncertain efficacy of grafted cells in the target organ is the main obstacle to cell therapy [11, 22–26]; thus, recent research has focused on developing new strategies to tackle this issue [22, 27, 28].

Hyaluronic acid (HA) is one of the most used biomatrices in human medicine, and multiple studies have suggested that it improves the engraftment efficacy of transplanted cells [9, 12, 18, 20–22, 29, 30]. Preclinical data have also highlighted some properties of HA that are promising for future applications in cell therapy of liver diseases. However, clinical applications of cell therapies are hindered by the lack of techniques that can track transplanted cells and verify their fate after injection.

In this review, first, we will summarize recent studies on HA and its cell receptor, cluster of differentiation 44 (CD44); second, we will give an overview of the use of HA in regenerative medicine and cell therapy; and lastly, we will discuss recent approaches to cell tracking with potential applications in humans.

## 2. Engraftment Efficiency and Factors Affecting Liver Engraftment

Human stem cell therapy is an active field of research. Understanding how to modulate the engraftment of transplanted or infused cells represents an important goal to improve the homing of grafted cells in the target organ and to minimize ectopic colonization. Although it has been hypothesized that cells cannot survive in ectopic sites, recent data from athymic mouse models have shown that cells can survive for months in ectopic sites, such as the lung, spleen, and kidney, and that they can be followed with positron emission tomography (PET) [22].

Several research groups are striving to find new strategies to reduce the ectopic localization of cells, and HA, a natural biomatrix found in most of the organs, is one of the most investigated molecules in the field of hepatology because of its multiple interesting properties [4, 9, 21, 31–36].

### 2.1. Cell Engraftment Efficiency

Experiments on different mouse models have shown that the highest liver engraftment efficiency of hepatic stem/progenitor cells was less than 5% when cells were transplanted via the intraportal route or other vascular routes [26, 37, 38]. Similar results were obtained by infusing stem cells via vascular routes into primate livers [26] or via the intraportal route in humans [24]; however, the engraftment efficiency in patients increased to 20-25% when the cells were infused through the hepatic artery [24].

Intrasplenic hepatocyte transplantation has been performed in animal models with chronic liver failure. After transplanting hepatocytes into the splenic parenchyma of rats, researchers observed a transient portal hypertension and noticed that approximately 26% of the cells remained in the spleen, 72% colonized the liver, and 2% were entrapped in the small capillaries of the lungs [26]. Recently, we have shown that transplantation via the intrasplenic route of HA-coated human biliary tree stem/progenitor cells (hBTSCs) in mice increased the engraftment efficiency by fivefold without significant cell distribution in ectopic sites [27]. It is important to point out that, after cell transplantation, grafted cells were present in nontarget organs [39], but in certain cases, most of the ectopic cells were no longer detectable after two days [40]. Liver parenchymal repopulation by exogenous cells is a prerequisite for successful cell therapies [41]. Cell translocation from sinusoids into liver plates requires the disruption of the sinusoidal endothelium and the progressive proliferation of the transplanted cells through a sequential process that involves chemokine-activated integrins and the ECM [42].

### 2.2. Factors Affecting Engraftment Efficiency

Several factors may affect the engraftment, such as the host characteristics and response, the cell source, and the administration route (see Box 1).

- Factors related to the host may be tissue vasculature, alterations in the blood system, pathologic conditions (necrosis, transmissible factors, inflammation, and fibrosis), and the ECM composition and structure (adhesion molecules, remodeling factors) [11, 22, 25, 39]
- Factors regarding the cell source may be cell size [41], cell proliferation [11, 43, 44], intrinsic immunogenicity [45–47], tolerance to toxic and ischemic injuries [45], metabolic/metabolomic cell properties [48], and the adhesion molecules associated with the cytoskeleton, whose expression is affected by both environment cues and the ECM [22, 28, 49]
- The engraftment efficiency depends also on the administration route. For instance, hepatic artery infusion and portal vein infusion lead to different engraftment levels [50]

Cell features associated with high engraftment efficiency include the aggregate size [41] and the cell size: cells with large size may cause venous thrombosis after transplantation and ischemia-related issues that can lead to loss of tissue functions. Regarding cell proliferation, the grafted cells should be able to proliferate more than the resident cells and acquire organ-specific physiological functions [11, 27, 44]. With regard to immunogenicity, the host's immune system should not be overstimulated to avoid rejection and toxic injuries in donor cells [45–47].

### 2.3. Strategies for Cell Delivery

Cell delivery techniques should maximize regenerative benefits while minimizing side effects [42]. In humans, both the portal vein and the hepatic artery are considered as safe administration routes in liver cell therapy. Although further comparative studies are needed to define the best delivery method [51, 52], both routes have so far shown complications, such as hepatic artery dissection following hepatic artery infusion [53] and increased portal hypertensive bleeding upon portal vein infusion [54].

Many ongoing studies are trying to improve the outcome of cell engraftment in the liver (Box 2). For instance, researchers are aiming to determine the best host preconditioning for hepatocyte cell therapy (i.e., the physiopathological conditions of the receiver before cell infusion) [39], the most appropriate matrix components to use (e.g., fibrin [26], cross-linked HAs [22, 28], or other biomatrix scaffold components [55]), the efficiency of direct injection as compared to vascular infusion (preclinical study) [22], or the efficacy of combination approaches (for instance, combining a grafting device with direct injection or transplanting recellularized liver scaffolds [56, 57]). However, there are no studies comparing how different cell types affect liver cell therapy.

## 3. CD44 as an HA Receptor

In human, the CD44 gene maps to the chromosomal locus 11p13. CD44 encodes for a glycoprotein involved in cell adhesion, and it is the best-characterized member of the hyaluronate receptor family.

### 3.1. CD44 Functions

Alternative splicing of the CD44 gene generates variants of the extracellular domain that confer different functions to the protein. The expression of variant isoforms has been observed in breast [58] and pancreatic ducts [59, 60].

CD44 binds to HA, and its activation is finely regulated. While the inhibition of N-glycosylation enhances HA binding, the mutation of specific sites converts the CD44 inducible form to the constitutively active form [61]. The receptor is involved in sensing the extracellular microenvironment and in intercellular cross-talk. CD44 proteins primarily maintain the 3D structure of organs and tissues and control the proliferation of epithelia and repairing of stressed cells. When cells expand on specific scaffolds, the expression of both CD44 and HA is enhanced [62, 63].

### 3.2. CD44 and HA

HA is the main ligand of CD44, and it is involved in cell-cell and cell-matrix adhesion, cell migration, and signaling. HA is a polymeric linear glycosaminoglycan that contains at least three sites that bind to CD44: a “link” domain encoded by exon 2 [64] and another two domains encoded by an overlapping region in exon 5 [65]. The HA binding sites consist of amino acid clusters that include specific arginine residues that mutation studies proved to be required for the binding [64, 66]. A detailed mutational analysis of amino acid clusters in the cytoplasmic domain of CD44 has identified specific arginine and lysine residues through which reagents stimulating protein kinase C (PKC) differentially regulate the binding of CD44 to HA [67]. Cells can express CD44 in an active, inducible, or inactive state depending on HA binding [61].

### 3.3. Other Ligands of CD44

Besides HA, CD44 binds to other ligands, including osteopontin, serglycin, collagen, fibronectin, and laminin, through its extracellular N-terminal domain, which is highly conserved (it displays about 85% homology among mammals) [68].

### 3.4. Other Receptors of HA

HA is also bound by hyaluronan-mediated motility receptor (RHAMM) and by lymphatic vessel endothelial receptor-1 (LYVE-1). RHAMM and CD44 are coexpressed, and RHAMM has similar but fewer functions than CD44 [69]. RHAMM promotes migration and proliferation of normal and tumor cells [69]. A recent study has suggested that LYVE-1 mediates leukocyte extravasation from lymphatic vessels [70].

## 4. Hyaluronic Acid

HA is expressed on the cell surface of both normal and tumor cells. It is an important component of the stem cell niches as it preserves the multipotency of stem/progenitor cells and prevents their differentiation; also, HA modulates stem cell migration during embryonic development [34].

For many years, the ECM was believed to have only mechanical properties; however, in the last decades, multiple studies have shown how the ECM plays a crucial and dynamic role in regulating cell homeostasis. Indeed, the HA matrix supports cell adhesion, growth, and differentiation, it regulates cell trafficking, and it affects various processes, such as development and organogenesis, inflammation, wound healing, and tissue remodeling [4].

Among the ECM components, HA has a crucial role because of its rheological, viscoelastic, and hygroscopic properties [4]. HA molecules interact with high efficiency and form large polymers in combination with other molecules, generating different complexes involved in cell motility, proliferation, adhesion, and differentiation [9]. HA can also adapt to variable three-dimensional configurations depending on pH, salt concentration, and associated cations. The HA complexes can form in highly viscous solutions with low concentrations of HA. By increasing HA concentration, solutions become more viscous since the linear polymers associate with each other forming bigger structures, which are stabilized by hydrophobic bonds between the chains [31]. HA prevents the accumulation of other macromolecules and delays the spread of contaminants and the migration of cells other than immune cells into tissues because of steric hindrance, the rotation around the linkages between sugar residues, and the dynamic and weak hydrogen bonds between the residues [31].

### 4.1. Anti- and Pro-inflammatory Properties of HA

Depending on the polymer length and the ability to bind to multiple CD44 molecules, HA can exert opposite functions. For instance, it can either promote or inhibit inflammation and fibrosis [9, 71]. CD44 binds to HA with low-affinity hydrogen bonds; as a result, multiple receptors need to bind to HA to trigger downstream signaling [72]. Two papers have suggested that the binding of CD44 to HA enhances T-cell antigen receptor (TCR) signaling leading to the activation of regulatory T-cell populations [73, 74]. Other authors have hypothesized that their binding induces the production of anti-inflammatory cytokines, such as IL-10 [71] and TGF-*β* [71, 75, 76], and it inhibits the pro-inflammatory Toll-like receptor (TLR) signaling and NF-*κ*B translocation [72]. However, the pathways through which CD44 enhances anti-inflammatory signals are unknown.

HA long-chains (HA-l) exhibit anti-inflammatory properties in many *in vitro* and *in vivo* models [35, 77]; studies have also reported that HA-l increase the phagocytosis by macrophages, reduce pro-inflammatory cytokine production, and limit cell oxidative damage and apoptosis [32, 36, 78].

HA short-chains (HA-s), which are generated by HA proteolysis, exhibit pro-inflammatory properties by modulating TLR-4 and TLR-2 signaling [79]. It has also been suggested that HA-s may play a double role during the inflammatory process by inducing both the expression of pro-inflammatory cytokine and TRL-4-mediated pathways [80]. A study by Saikia et al. [81] supports this hypothesis: the authors found that the miRNA miRNA-181b-3p was downregulated in Kupffer cells of alcoholic liver disease (ALD) patients. This miRNA dampens inflammation by inhibiting the expression of importin *α*5, which activates NF-*κ*B. Interestingly, treating Kupffer cells with hyaluronic acid 35 (HA35), a small specific-sized HA, restored the expression of miRNA-181b-3p. Indeed, HA-s makes the ECM more accessible to immune cells and induces pro-inflammatory pathways in the surrounding cells that, in turn, release cytokines that attract more immune cells [82].

## 5. Biologic Rationale for the Use of HA and Its Derivatives in Regenerative Medicine

Currently, HA is one of the most important molecules used to craft biomaterials, and it has been employed in different areas because of its multiple roles [9].

### 5.1. HA Modifications

The carbohydroxilic groups of HA can be modified generating two main groups of molecules by covalent cross-linking of native HA. The first group is created by a reaction that requires toxic reagents and harsh conditions and that makes the resulting hydrogel unable to bind to tissues and cells. On the other hand, the second group can be further modified and is able to interact with cells, tissues, and therapeutic agents. Therefore, this second type of HA derivatives is useful for clinical studies [83].

### 5.2. Tyramine-Modified HA

Recently, a tyramine-modified HA has been generated by in situ enzymatic cross-linking by adding hydrogen peroxide to solutions of HA-tyramide; further developments will allow using tyramine-modified HA for cell delivery [84]. Tyramine-modified HA can form hydrogels that can modulate, *in vitro* and *in vivo*, cellular mechanisms such as delivery, recovery, and expansion.

### 5.3. Thiol-Modified HA

Thiol-modified HA, used for drug evaluation and regenerative medicine, is obtained by modifying the carbohydroxilic groups through hydrolysis of the disulfide bonds [19, 85]. The biodegradation rate and specific mechanical properties of thiol-modified HA, such as physical form, viscosity, and transparency, can be modified [86]. For instance, the aldehyde-modified HA has been proposed for vocal fold wound healing because of its adjustable viscoelasticity conferred by the double cross-linked networks between HA microgels and cross-linked hydrogels [9]. Shu et al. investigated the potential application of the thiol-modified HA in tissue repair by using a range of HA concentrations between 1.0 (*w*/*v* %) and 0.0 (*w*/*v* %) in order to obtain different levels of hydrogel stiffness; they observed that cells proliferated better in thiol-modified HA hydrogels than in culture dishes [85].

### 5.4. Mixing HA and Soluble Signals

Turner et al. have studied how to improve liver engraftment of human hepatic stem cells (hHpSCs) by using a mix of soluble signals and extracellular matrix biomaterials that are found in stem cell niches (hyaluronans, type III collagen, and laminin) [22, 28]. In their works, Turner and colleagues used different HAs with high molecular weight (average MW: 1,500,000), and they diluted them to obtain a range of final concentrations of 1.0 and 2.0% solution (*w*/*v*).

Recently, a functional wound dressing composed of different biomaterials, including HA and collagen, and containing epidermal growth factor (EGF) and vitamin C derivative (VC) has been developed [18, 30, 87, 88]. Niiyama and Kuroyanagi investigated the properties of this wound dressing as a cultured dermal substitute (CDS), its potential to facilitate the production of vascular endothelial growth factor (VEGF) and hepatocyte growth factor (HGF) *in vitro*, and its ability to enhance granulation tissue formation associated with angiogenesis and collagen deposition *in vivo* [18].

### 5.5. Parameters of HA Hydrogels

Both the composition and the mechanical properties of the microenvironment in which cells are seeded are key factors to control the cell phenotype and differentiation. Lozoya et al. discovered that they were able to guide the differentiation of human hepatic stem cells by changing the HA concentration from 1.0 to 2.0 (*w*/*v* %) in hydrogels [49]. Their results may be useful to find new strategies to expand and differentiate stem/progenitor cells isolated from soft organs. The mechanical and biochemical properties of cells embedded in a matrix can be analyzed separately; the combination of these properties guides the design of parenchymal tissues for cell therapies and the development of bioreactors [49].

The most important benefit of HA is its biocompatibility [13, 85, 89]; indeed, Shu et al. have shown that it facilitates tissue regeneration in nude mice [85]. HA is an attractive candidate for stem cell grafting because of its abundance in embryogenesis, wound repair, and organ regeneration [49]. However, a good matrix for tissue engineering needs to replicate both the biochemical and the mechanical properties of the environment where the cells are transplanted [20, 21, 90]. Lozoya and colleagues studied hHpSC grafting by using a range of concentrations of hydrogel with thiol-modified HA derivatives (CMHA-S) and polyethylene glycol diacrylate (PEGDA) as a cross-linking agent. They demonstrated that both composition and mechanical properties of the microenvironment regulate cellular phenotypic changes; furthermore, their model allows studying stem cell functions in 3D cultures [49].

### 5.6. HA Hydrogels and Gels for Cell Delivery in Non-hepatic Tissues

Chang et al. used human HA hydrogels to transplant epicardial stem cells (they used high molecular weight HA at a final concentration of 10% *w*/*v*) [12]. The authors decided to test HA-blood hydrogels because they are easy to synthesize, promote stem cell survival and proliferation, and are promising candidates for cell delivery in the epicardium [12]. Compared to HA or PEG-based gels, HA-blood hydrogels offer the possibility of synthesizing hydrogels with autologous blood, which is important for a potential application into the clinic [6, 29]. Combining autologous blood and HA has several advantages as both components provide adhesion motifs that activate prosurvival pathways [91]. Blood contains vitronectin and fibronectin with arginine-glycine-aspartate motifs that activate integrins, and HA receptors (CD44), which are expressed by many stem cells. Moreover, blood provides growth factors to the transplanted cells before new vasculature is established, and HA and its degradation products promote angiogenesis, vasculogenesis, and cardiogenesis [20]. Hydrogels can be degraded by enzymes such as hyaluronidases and proteases and also by hydrolysis. Covalent cross-linking allows HA-blood hydrogel synthesis, and adhesion to transplanted tissue without using ultraviolet light, heat, or sutures can facilitate their clinical translation [12]. Although pioneering, the study by Chang et al. is limited by the fact that the researchers evaluated the survival and the proliferation of cardiosphere-derived cells only *in vitro*. Moreover, the authors suggest that additional studies on different types of stem cells and hydrogels are needed to assess the efficacy of blood-HA hydrogels in small and large animal models [12].

Dietrich et al. analyzed the engraftment efficiency of human adipose-derived stem cells (ADSCs) in HA gel when subcutaneously injected in athymic mice [14]. The vasculature that developed in the ADSC implants for two months was probably supported by the paracrine interaction between ADSCs, host ECM, and endothelial cells, and it was induced by the proangiogenic signals released by HA degradation. The authors hypothesized that ADSCs promote angiogenesis by secreting chemotactic cytokines that attract endothelial cells. Another factor that may have contributed to the vascularization of the implants is the secretion of hyaluronidase by ADSCs, which leads to the release of HA fragments [14].

Altman et al. implanted in a photoaged skin murine model ADSCs seeded into a new-generation HA preparation: the nonanimal stabilized HA, an HA entirely produced from nonanimal sources, which provides an organized fibrovascular network able to support the implants [7].

## 6. Cell Tracking

Cell labeling and tracking are important tools to understand the biological mechanisms behind cell engraftment and verify the therapeutic effects of inoculated cells *in vivo*. Indeed, they allow analyzing cell behavior, engraftment efficiency, cell localization, and cell fate. Recent negative clinical trials have highlighted the need for new noninvasive methods of cell tracking and markers of cell engraftment efficiency for liver cell therapy, especially in cases of liver cirrhosis and acute liver failure [92, 93].

Many approaches, either direct or indirect, have been developed to visualize engrafted cells *in vivo* and to distinguish transplanted cells from host cells [7, 94–100]. In direct labeling approaches, the target cells are labeled with probes prior to transplantation but, once inoculated, the biological environment hampers their tracking even with appropriate imaging equipment. To overcome these limitations, researchers have developed indirect labeling methods that involve genetic modifications to tag and track cells [101–103]. However, using either nonviral or viral vectors to mutate genes may increase the risk of uncontrolled gene expression and, therefore, of tumor formation [104, 105].

### 6.1. Tetra-acetylated N-Azidoacetyl-D-mannosamine Cell Labeling

Kang et al. introduced an innovative tracking strategy *in vivo* based on bioorthogonal chemical reporters [106]. First, they treated cells with tetra-acetylated N-azidoacetyl-D-mannosamine (Ac4ManNAz) to induce the expression of unnatural azide-modified sialic acids on the surface of target cells. Ac4ManNAz has high reactivity and low toxicity [107–110], and it does not affect cell viability [111]. However, it is worth mentioning that studies have reported that sialic acids may affect cell adhesion, cell-cell interactions, and migration [107, 108, 110]. After transplanting the cells into the livers of nude mice, Kang and colleagues injected intravenously dibenzylcyclooctyne-conjugated Cy5 (DBCO-Cy5) to visualize the target cells *in vivo*. By using this strategy, the authors were able to enhance labeling efficacy and facilitate cell tracking. Moreover, they reduced the false positive signal caused by macrophages engulfing engrafted cells since the macrophages did not express azide groups after phagocytosis [111].

### 6.2. Using Nanoparticles for Cell Labeling

In order to track cell engraftment, some research groups have used nanoparticles (NPs) and visualized them with magnetic resonance imaging (MRI). Since protein-based NPs cannot be imaged by MRI because they do not generate enough contrast, they need to be labeled with paramagnetic or superparamagnetic nanomaterials (magnetically labeled nanoparticles (MLNPs)) [112]. Vera et al. have shown that MLNPs can be traced by MRI in a rat's brain. Even though they used a clinical MRI machine with limited sensitivity, they were able to detect the diffuse and global accumulation of MLNPs by implementing a new histogram technique [113].

MRI was used to detect the presence of labeled human cells transplanted into the liver of murine and rat models. The proposed method may be used to monitor the engraftment of any types of cells in any animal models.

### 6.3. Supermagnetic Iron Oxide Cell Labeling

MRI of superparamagnetic iron oxide- (SPIO-) labeled cells is a sensitive and noninvasive method that allows tracking of cell populations inside the brain [114–119], bone marrow [120–122], kidneys [123, 124], and myocardial tissue [125–127]. SPIO is considered as a promising labeling agent for *in vivo* cell tracking because it maximizes the spatial resolution of MRI; moreover, as SPIO causes a strong susceptibility effect, it allows the detection of small amounts of labeled cells [128, 129].

Wang et al. tracked and quantified with MRI SPIO-labeled endothelial progenitor cells (EPCs) after transplantation into murine injured livers [130]. The results indicated that the relaxation rates R2 and R2^∗^ depended on the number of cells that were labeled *in vitro* before injection; therefore, the authors suggested that measuring the relaxation rates, and R2^∗^ in particular, may help to quantify cell homing *in vivo* and be useful parameters to take into account for cell transplantation therapies [130].

### 6.4. Cell Labeling with Antibody-Conjugated Magnetic Microbeads

McClelland et al. tracked *in vivo*-transplanted human hepatic stem/progenitor cells (hHpSCs and hHBs) by labeling them in situ with magnetic microbeads conjugated to an antibody against a surface antigen that is expressed only by hepatic progenitors. The labeled cells were imaged both in NOD-SCID mice and in Sprague-Dawley rats [131].

### 6.5. Quantum Dot Cell and Qtracker Labeling

Another method to track cells is based on fluorescent nanoparticle quantum dots (Qdots). These nanoparticles are excellent tools for long-term tracking and imaging studies of living cells. For example, Carpino et al. used Qtracker, a labeling system based on Qdots, to isolate from human gallbladder cells expressing the Epithelial Adhesion Molecule (EpCAM). They noticed that the fluorescent nanocrystals were passed on to daughter cells after replication without the need for a specific enzyme. Moreover, the isolated cells showed properties typical of stem cells, such as clonogenic proliferation [132].

Lin et al. studied mobility, viability, proliferation, and fusion of mouse embryonic stem cells by tagging them with different Qtrackers (525, 565, 605, 655, 705, and 800) in mouse models. They concluded that the labeling system did not affect viability, proliferation, or differentiation potential of stem cells, and they were able to detect Qtracker signals after injecting labeled stem cells into athymic mice [133]. Another benefit of using Qtrackers is their size: as they are larger than organic dyes, they cannot spread between cells, unless the cells undergo cell fusion. For this reason, they are excellent tools to study cellular interactions [134].

In conclusion, HA is of crucial importance for both the ECM and *in vitro* scaffold matrices used for cell growth. Modifying HA to generate hydrogels that can modulate intra- and intercellular processes opens the way to pioneering therapies. However, in order to verify the benefits of cell therapy, HA scaffolds must be coupled with labeling systems that allow characterizing cells *in vitro* and tracking them *in vivo* after inoculation.

## 7. HA-Coated hBTSCs as Potential Therapeutic Agents

The anti-inflammatory effect and the biocompatibility of HA are among the main benefits of using this molecule for liver engraftment [33, 85, 135, 136]. Indeed, it has been shown that different forms of HA limit fibrosis and foster vascularization in transplantations and that it can promote engraftment in mice [85]. HA is also a good candidate for stem cell grafting because of its abundance in embryogenesis, wound repair, and organ regeneration. Importantly, HA is available in a version that complies with cGMP manufacturing requirements and is approved for clinical use (in particular, for osteoarticular, cartilage, and cutaneous inflammatory damages) [13, 89]. Moreover, 90% of HAs are actively cleared by the liver [22, 135, 137].

**Figure 1:**
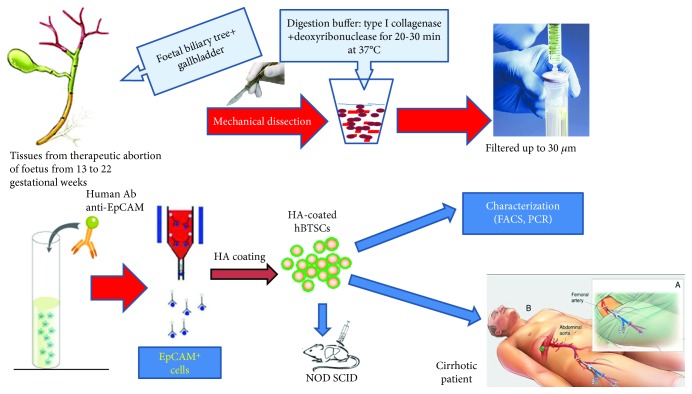
Our proposal for a cell therapy to treat cirrhotic patients. The figure shows a schematic representation of our protocol to treat cirrhotic patients that are not eligible for orthotopic liver transplantation.

We have recently demonstrated that injecting HA-coated hBTSCs into the liver increases cell engraftment; our technique is simple, feasible, and clinical-grade, and it meets all requirements for a fast transition from bench to clinical application (Figure 1) [27]. On the other hand, transplantation by direct injection or via a vascular route resulted in inefficient engraftment and cell spreading to ectopic sites [27]; similar results were obtained in previous studies that tested fibrin coating [22, 28, 79].

## 8. Conclusion

Cell therapy is an innovative approach to treat advanced liver diseases. It is of particular importance to understand the factors that regulate cell engraftment into the liver, such as cell-cell and cell-ECM interactions, cell proliferation, and immunogenicity, as well as to define the best transplantation routes. Several strategies have been developed to increase the efficiency of engraftment, and many of them are based on HA hydrogels with or without chemical modifications that can improve its biological properties. The use of HA in preclinical studies has led to promising results because of its biocompatibility and its role in regenerative processes. Moreover, several authors have shown the potential of cell tracking as a helpful tool in determining cell localization and engraftment rate. However, further studies are required to improve engraftment efficiency and move forward into clinical trials.

## Figures and Tables

**Box 1:**
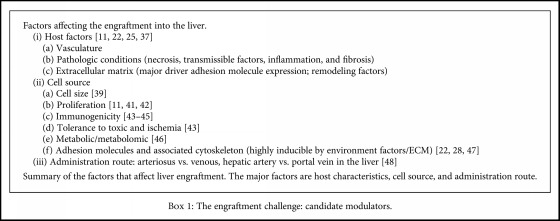
The engraftment challenge: candidate modulators.

**Box 2:**
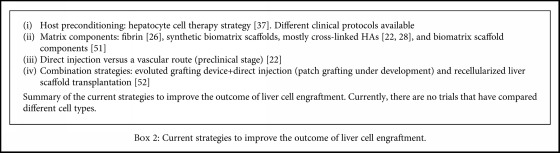
Current strategies to improve the outcome of liver cell engraftment.

## Abbreviations

ADSC: Adipose-derived stem cell
CAM: Cell adhesion molecule
CD: Cluster of differentiation
CMHA-S: Thiol-modified hyaluronic acid derivatives
Col: Collagen
CSD: Cultured dermal substitute
ECM: Extracellular matrix
EGF: Epidermal growth factor
HA: Hyaluronic acid
HA-l: Hyaluronic acid long-chain
HA-s: Hyaluronic acid short-chain
hBTSC: Human biliary tree stem/progenitor cell
HGF: Hepatocyte growth factor
hHpSC: Human hepatocyte stem cell
MLNP: Magnetically labeled nanoparticle
MR: Magnetic resonance
MRI: Magnetic resonance imaging
NP: Nanoparticle
PEGDA: Polyethylene glycol diacrylate
PET: Positron emission tomography
PKC: Protein kinase C
Qdot: Quantum dot
SPIO: Superparamagnetic iron oxide
TCR: T-cell antigen receptor
TLR: Toll-like receptor
VC: Vitamin C derivate
VEGF: Vascular endothelial growth factor.
